# Primary hepatic osteosarcoma: a case report and literature review

**DOI:** 10.3389/fonc.2025.1669395

**Published:** 2025-12-17

**Authors:** Haiping Luo, Hongmin Yu, Gaochun Xiao

**Affiliations:** 1 Department of Gastrointestinal Surgery, Huangshi Central Hospital, Affiliated Hospital of Hubei Polytechnic University, Huangshi, China; 2 Department of Breast and Thyroid Surgery, Huangshi Central Hospital, Affiliated Hospital of Hubei Polytechnic University, Huangshi, China; 3 Department of General Surgery, Taihe Hospital, Hubei University of Medicine, Shiyan, Hubei, China

**Keywords:** osteosarcoma, extra-skeletal, liver, pathological diagnosis, case report

## Abstract

**Background:**

Extra-skeletal osteosarcoma (ESOS) is an uncommon malignant soft tissue tumor, primarily seen in the soft tissues of the extremities or the retroperitoneal region. Primary hepatic osteosarcoma (PHO), a unique subtype, is clinically exceedingly rare. Thus far, only isolated instances have been documented in the literature, with limited high-quality study data accessible. Furthermore, there is no definitive clinical consensus regarding the ideal characterization and management of PHO.

**Case presentation:**

This case report details a 67-year-old male patient hospitalized for one month due to abdominal pain. Upon admission, the patient displayed an increased serum alkaline phosphatase level. Computed tomography (CT) and magnetic resonance imaging (MRI) identified a well-defined lesion in the left hepatic lobe. The patient underwent an open left hemihepatectomy to obtain a conclusive diagnosis. Postoperative histological and molecular pathology assessments verified the tumor as a PHO. The patient underwent transhepatic arterial chemotherapy with epirubicin 30mg/m² and cisplatin 40mg/m², succeeded by 3 cycles of MAP regimen chemotherapy (methotrexate, doxorubicin, cisplatin) in conjunction with sorafenib-targeted therapy, achieving a recurrence-free survival (RFS) of 21 months.

**Conclusion:**

With a median age upon presentation of 61 years, PHO primarily affects men. It is identified radiologically by cystic-solid tumors, sometimes accompanied by calcifications, which facilitates early radiological detection. The literature analysis and our case report point to TP53 mutations and aberrant SATB2 expression as possible genetic markers that could close the diagnostic gap for this uncommon and frequently misdiagnosed illness. Preliminary findings indicate that multimodal therapies—surgery, chemotherapy, and targeted therapy—hold promise for improving patient survival despite PHO’s high malignancy and poor prognosis.

## 1 Introduction

Osteosarcoma is a common primary malignant bone tumor that develops from mesenchymal cells that create bones. It generally develops in the metaphysis of long bones, especially the distal femur, proximal tibia, and proximal humerus (1). However, extra-skeletal osteosarcoma (ESOS) is still quite uncommon, nevertheless, and primary hepatic osteosarcoma (PHO) is a very uncommon condition with very few examples reported in the literature (2, 3).

PHO is extremely rare, and its nonspecific clinical symptoms make diagnosis and treatment extremely difficult. Hepatomegaly, weight loss and abdominal discomfort are prominent symptoms in affected patients that overlap with those of prevalent hepatic malignancies (3, 4). There are currently no defined standardized treatment procedures for PHO because the underlying etiology is still poorly understood (5). These difficulties are exacerbated by the fact that the majority of the information currently available about this illness comes from individual case reports, which do not have the backing of extensive research required to offer solid evidence-based clinical guidance (4).

In this report, we describe a rare instance of PHO in a 67-year-old male patient. Our aim in reporting this case is to contribute to the expanding body of literature on this uncommon cancer and to improve clinical awareness and understanding among healthcare professionals.

## 2 Case description

A 67-year-old male appeared to our outpatient clinic with a one-month history of stomach pain and distension. The patient exhibited symptoms starting in September 2023, characterized by a progressive emergence of right upper quadrant stomach pain, unaccompanied by fever, chills, acid reflux, belching, nausea, vomiting, or jaundice of the skin and sclera. Furthermore, he indicated a diminished appetite and an inadvertent weight loss of approximately 3 kg.

The patient’s comprehensive medical history encompasses a right nephrectomy performed via an open operation over 30 years prior, untreated chronic hepatitis B persisting for the same duration, and a 10-year history of liver cirrhosis. Furthermore, he has been treating chronic obstructive pulmonary disease untreated for almost 8 years. The chronic diseases, especially the liver-related disorders, indicate a complicated interaction of elements that may be contributing to his current stomach complaints, necessitating a thorough evaluation.

The patient had indications of chronic condition and significant emaciation. No jaundice was observed in the skin or conjunctivae, and there were no palpable superficial lymph nodes present. The abdomen was non-tender and pliable, exhibiting no signs of rebound tenderness or masses. Edema of the lower limbs was not observed.

## 3 Diagnostic assessment and treatment

### 3.1 Laboratory examinations

Standard laboratory examinations, encompassing blood, urine, and stool analysis, indicated no notable abnormalities. With respect to serum parameters, the concentrations of direct and indirect bilirubin were within the normal range. Conversely, the concentrations of alkaline phosphatase (ALP), Hepatitis B viral DNA (HBV DNA), and carbohydrate antigen 125(CA 125) were markedly increased.

Moreover, the concentrations of tumor markers, namely carcinoembryonic antigen (CEA), carbohydrate antigen 19-9 (CA 19-9), carbohydrate antigen 72-4 (CA 72-4),and alpha-fetoprotein (AFP),were all within the reference range, details are shown in **Table 1** (6).

**Table 1:** Laboratory test results summary.

Variables	reference range	test result
Direct bilirubin (μmol/L)	0-8.0	6.0
Indirect bilirubin (μmol/L)	2.8-13.2	10.0
ALP (U/L)	45-125	341
HBV-DNA (IU/mL)	<20	47.29
CEA (ng/mL)	0-5.09	2.39
CA 125 (IU/mL)	0-35	126.60
CA 19-9 (IU/mL)	0-37	8.87
CA 72-4 (U/mL)	0-6	4.56
AFP (ng/mL)	0-7	5.0

### 3.2 Imaging findings

**Figure 1:**
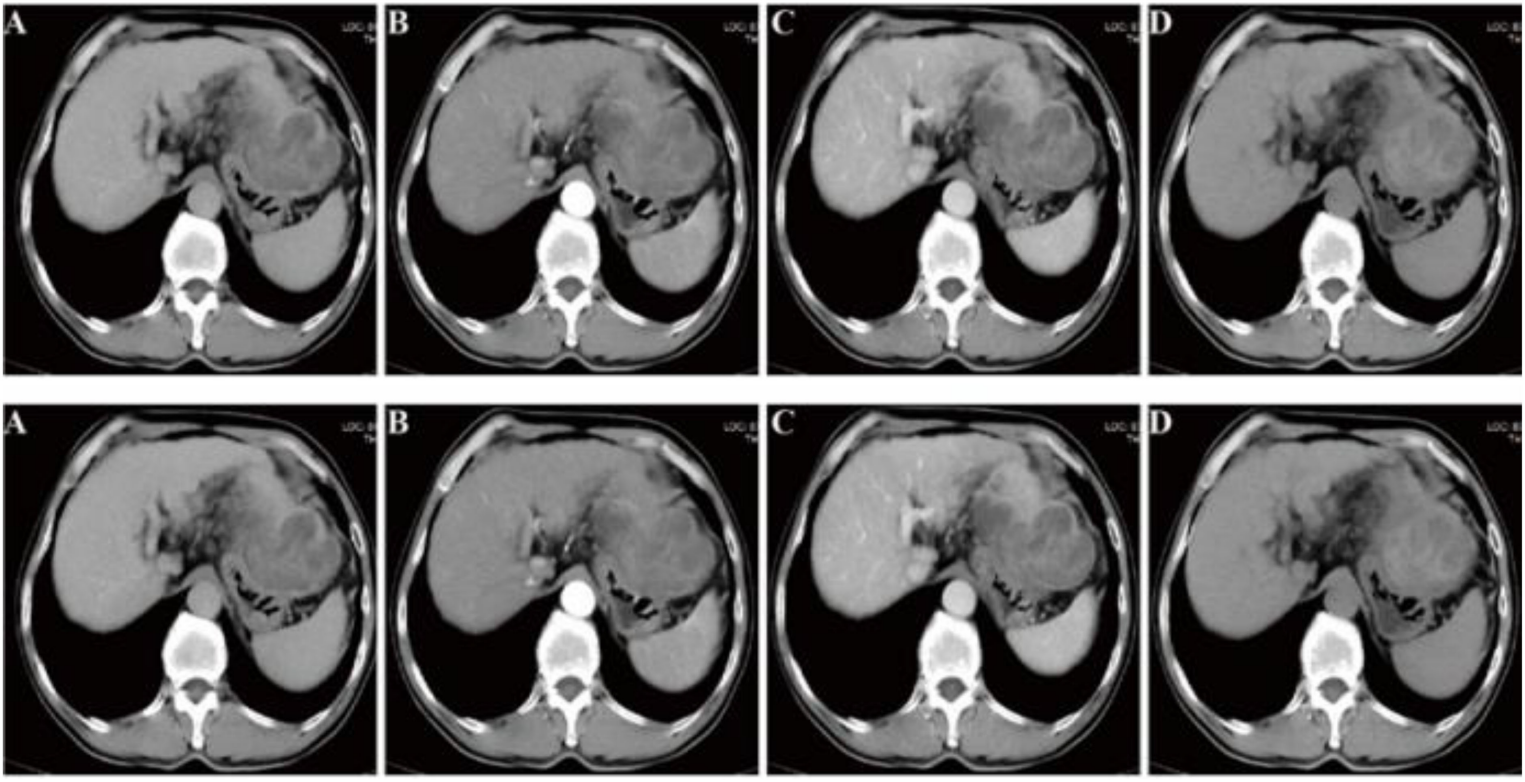
CT findings: **(A)** Plain CT scan reveals a sinuous appearance of the hepatic margin and an unusual mass in the hepatogastric region. **(B)** The tumor receives blood supply from the left hepatic artery, exhibiting flocculent increase during the arterial phase. **(C, D)** The tumor exhibits heterogeneous enhancement during the venous phase and delayed phase.

Abdominal contrast-enhanced computed tomography (CT) revealed an undulating hepatic contour and an irregular mass lesion in the hepatogastric ligament, characterized by vascular supply from the left hepatic artery (**Figure 1A**). The lesion exhibited punctate arterial-phase enhancement during the arterial phase, along with diverse enhancement patterns in the venous and delayed phases of contrast imaging (**Figures 1B–D**).

**Figure 2:**
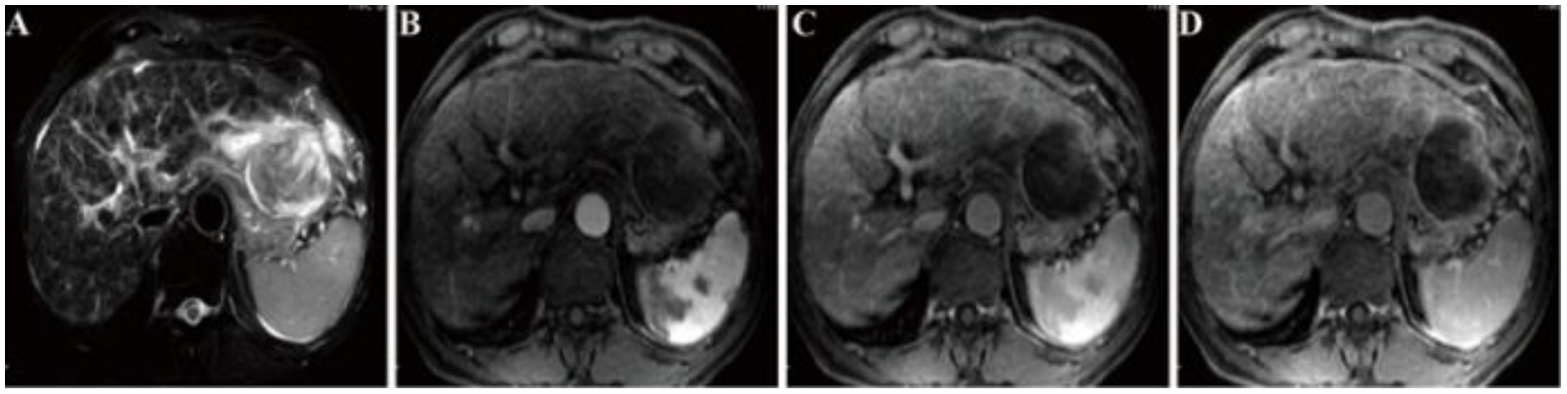
MRI findings **(A)** An irregular mass with a size of approximately 7.5 cm × 6.9 cm is observed in the left lobe of the liver, exhibiting heterogeneous hyperintensity on T2WI. On contrast-enhanced MRI, the tumor exhibits heterogeneous enhancement during the **(B)** arterial phase, **(C)** venous phase, and **(D)** delayed phase.

Contrast-enhanced magnetic resonance imaging (MRI) of the liver, incorporating diffusion-weighted imaging (DWI), demonstrated an uneven hepatic contour and heterogeneous hypointensity on T2-weighted imaging (T2WI). An irregular mass, measuring approximately 7.5 cm×6.9 cm, was identified in the left hepatic lobe (**Figure 2A**); this mass presented mixed hyperintensity on T2-weighted imaging (T2WI), restricted diffusion on DWI, and heterogeneous enhancement following contrast administration (**Figures 2B–D**).

### 3.3 Surgical findings and pathological examination results

**Figure 3:**
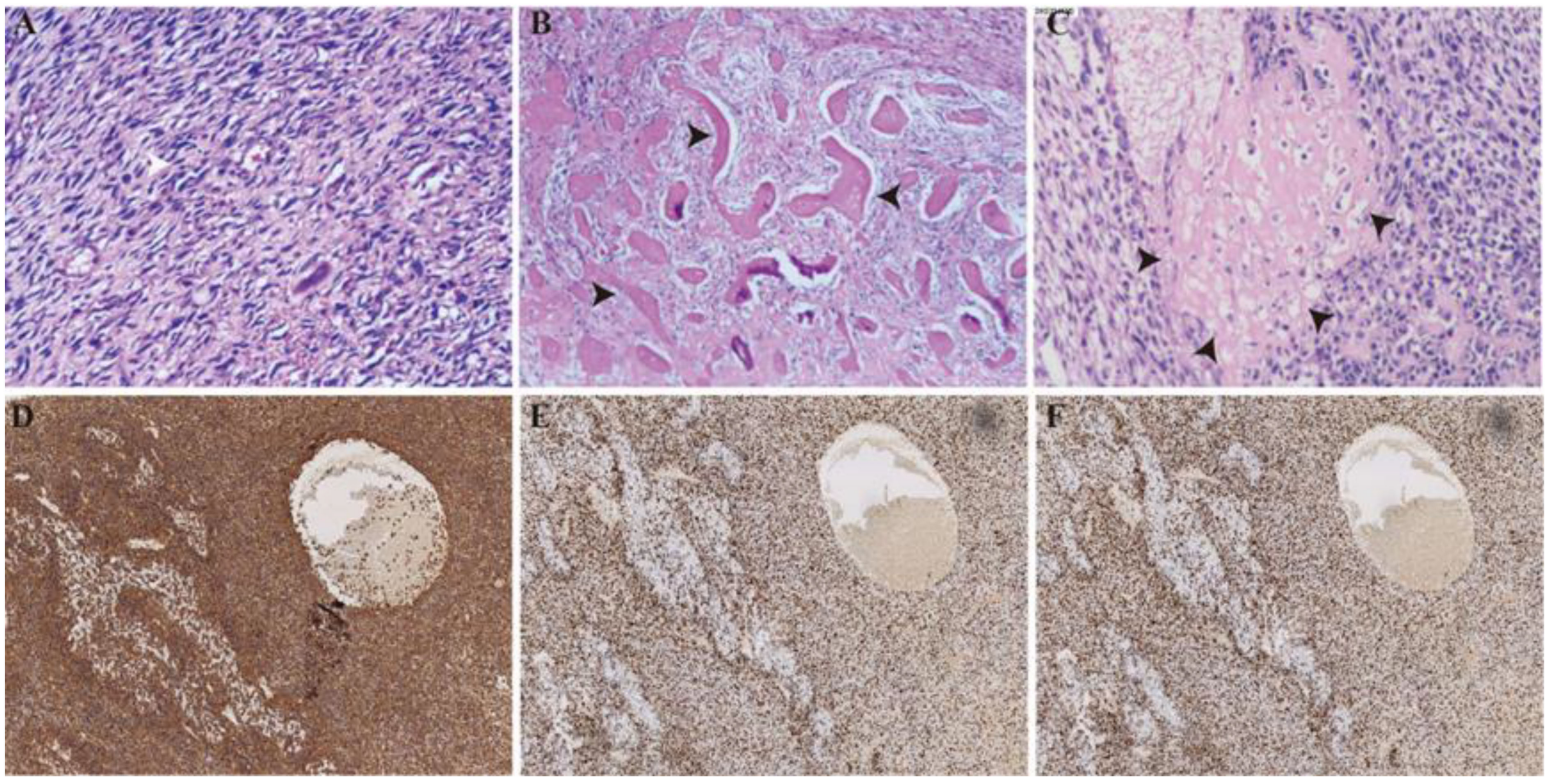
Histologic and immunohistochemical features of the tumor. **(A)** The neoplasm mostly consists of spindle cells, epithelioid cells, and clear cells (H&E stain, original magnification,100×). **(B)** Osteoid matrix (black arrows) (H&E stain, original magnification,40×). **(C)** Chondroid matrix (black arrows) (H&E stain, original magnification,100×). **(D)** The tumor cells exhibited positivity for Vimentin (immunohistochemistry, original magnification,100×). **(E)** The tumor cells exhibited positivity for Ki-67 (immunohistochemistry, original magnification,100×). **(F)** The tumor cells were positive for SATB2 (immunohistochemistry, original magnification,100×).

In October 2023, the patient underwent an open left hepatectomy, which identified an 8.5cm×7.5cm mass within the left hepatic lobe. Pathological analysis showed that the tumor, which was mostly composed of spindle, epithelioid, and clear cell components (**Figure 3A**) along with sporadic tumor giant cells and mitotic patterns, had invaded the liver margins. Significant bleeding and necrosis were present, along with focal osteoid (**Figure 3B**) and chondroid matrix (**Figure 3C**), a robust vascular supply, and indications of neoplastic osteogenesis. The mitotic count in the hot spot of the tumor is 23/50 HPF. The immunohistochemical analysis revealed that the tumor had negative staining for CK(pan), CD117, Dog-1, CD34, S-100, CD31, MDM2, CDK4 and SOX-10. In contrast, strong positive staining was observed for SMA, Vimentin, and INI-1.Furthermore,the Ki-67 proliferation index was significantly increased, nearing 90%, with ERG exhibiting localized positive and SATB2 demonstrating diffuse positivity (**Figures 3D–F**).High-throughput sequencing indicated that the tumor is microsatellite stable (MSS),with a KRAS gene mutation in exon 3 (c.182A>T, p.Q61L) present at 41.50%,and a TP53 gene mutation in exon 8 (c.818G>A, p.R273H) at 50.95%.

### 3.4 Postoperative treatment and follow-up

**Figure 4:**
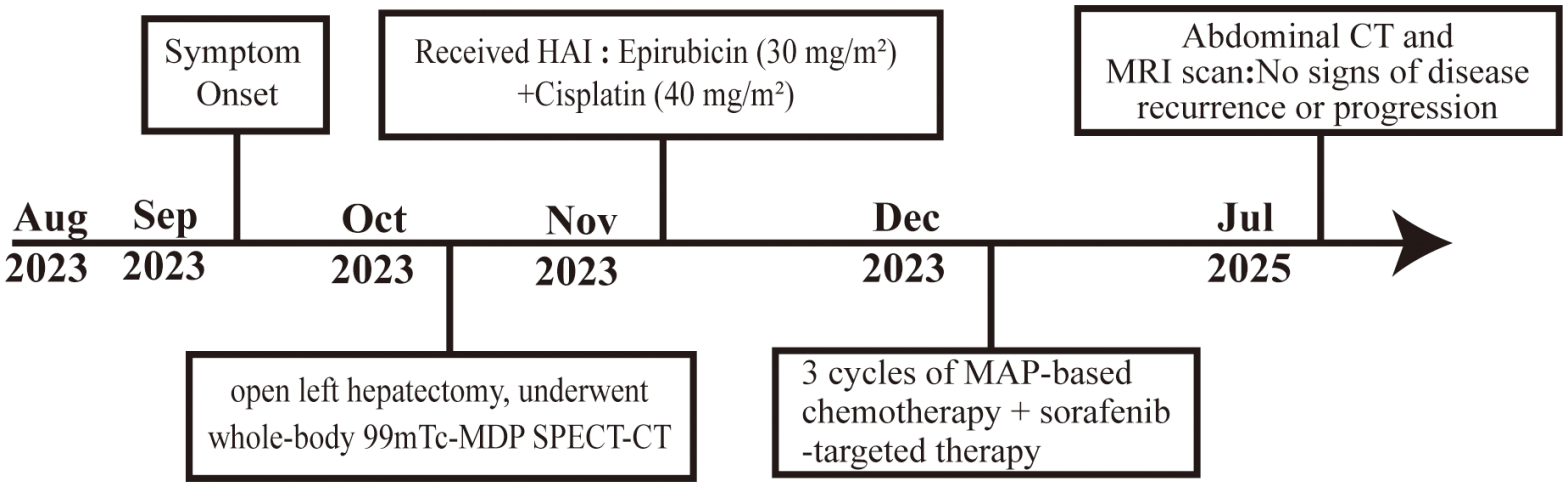
The patient’s timeline.

Postoperatively, the patient refused PET/CT due to financial limitations and had whole-body 99mTc-MDP SPECT-CT, which revealed no abnormalities and indicated PHO. Post-surgical liver function tests indicated a normalization of ALP, specifically 67U/L. Shortly after surgery, within 2 weeks, the patient underwent transhepatic arterial infusion chemotherapy (with epirubicin at a dose of 30 mg/m² and cisplatin at a dose of 40 mg/m²). Subsequently, 3 cycles of MAP (methotrexate, doxorubicin and cisplatin)-based chemotherapy plus sorafenib-targeted therapy were administered. Up to the present, in July 2025, as of the latest evaluations, the patient remains free of symptoms, and both CT and MRI imaging confirm no signs of disease recurrence or progression, the care timeline is shown in **Figure 4**.

## 4 Discussion

ESOS is an extremely rare mesenchymal neoplasm, usually occurring in the soft tissues of the extremities and retroperitoneum, with hepatic involvement being particularly uncommon (2, 7). Given its provision of key data on PHO’s clinical traits, diagnostic methods, and treatment results, this case carries substantial significance. **Supplementary Table S1** offers a thorough compilation of clinical features and treatment regimens from 26 prior PHO reports, including the current case (3–5, 8–29). The results of the reviewed studies indicates that the median age of onset for PHO is 61 years, consistent with the attributes of ESOS. Unlike the gender-neutral occurrence of ESOS, PHO demonstrates a significant male predominance. Research indicates that younger ESOS patients exhibit a better outcome. In accordance with this observation, a previously documented case detailed a 19-year-old female patient with ESOS who attained a prolonged survival duration of 36 months (17).

The size and location of the tumor are connected to the clinical symptoms that individuals with HPO experience. The most common nonspecific symptoms of PHO patients are distension and stomach pain, which are frequently missed and misinterpreted. Compared to ESOS with a tumor diameter of less than 5cm, whereas individuals with tumors surpassing 10cm demonstrate an even more unfavorable outcome (30, 31). As per the literature, all tumors exceeded 5cm, with the overwhelming majority exceeding 10cm—findings that indicate a high degree of malignancy and rapid progression in PHO (3–5, 8, 10, 14–19, 22, 25, 28). Patients with PHO often possess concomitant viral hepatitis and liver cirrhosis, resulting in hepatic inflammation and hypoxia (8, 9, 12, 13, 19, 22, 23, 26). This clinical condition may promote the advancement of PHO through various pathways, including connective tissue metaplasia, reactivation from embryonic dormancy, and osteogenic cell migration (3, 28).

Serum tumor markers in PHO, such as CEA, CA125, CA199 and AFP, may present as either normal or high, exhibiting a lack of specificity. Unlike primary hepatocellular carcinoma, most PHO patients present with normal serum AFP levels, with only two individuals demonstrating an increase. This discovery is significant for differential diagnosis (4, 5, 13, 15, 16, 18, 21, 24, 25, 28, 29). Simultaneously, the increase in blood ALP in PHO patients typically normalizes post-surgery, aiding in the exclusion of osteosarcoma at other locations and potentially serving as a potential biomarker for the completeness of PHO resection.

The results of the reviewed studies indicate that among 23 PHO patients with documented tumor locations, radiological assessments revealed a predominant occurrence in the right hepatic lobe (18 cases, approximately 78.3%).

(3, 4, 8, 9, 13, 15, 16, 18–22, 24–29). Within our patient sample,21 individuals underwent CT scanning, revealing that PHO typically has a cystic morphology with dispersed solid components. Notably, calcifications were observed in 17 of these 21 patients, accounting for over 50% of the total instances and approximately 81.0% of those who received CT scanning. This characteristic, although not exclusive to PHO, aids in differentiating PHO from other calcified hepatic tumors, including hepatic cystadenocarcinoma, cholangiocarcinoma, and metastatic lesions (29). Post-contrast CT and MRI scans demonstrate heterogeneous tumor enhancement, a characteristic that, although not unique to PHO, contributes to the diagnostic criteria. Given the scarcity of PHO, the utilization of whole-body 99mTc-MDP SPECT-CT and PET-CT is essential. These imaging methods facilitate the distinction between liver metastases originating from extrahepatic primary osteosarcomas and PHO, thereby allowing for precise diagnosis and staging. This distinction is essential for directing suitable treatment approaches and evaluating prognosis (27).

Upon microscopic examination, PHO is characterized by the tumor’s synthesis of osteoid, bone, or cartilage-like extracellular matrices, which are differentiated from bone and periosteal tissues (3). Although tumor cells may have vimentin expression, this marker lacks specificity for PHO (32). The conclusive identification of PHO is aided by the presence of SATB2 protein within tumor tissues (33). The Ki-67 index in PHO tumor samples often surpasses 10%, indicating elevated tumor cell proliferation, heightened recurrence and an overall poor prognosis (34). Mutations in the TP53 gene are frequently observed in osteosarcomas and are essential for molecular pathological diagnosis (35). The findings from the literature review indicate that TP53 gene mutations were present in all patients who underwent genetic testing, further validating the diagnostic utility of TP53 mutations for PHO. Thus, the combined assessment of SATB2 protein expression and TP53 gene mutation status is crucial for differentiating PHO from other hepatic malignancies, including hepatic cystadenocarcinoma, cholangiocarcinoma and carcinosarcoma.

PHO is extremely aggressive and advances swiftly, with untreated individuals frequently succumbing within one month of symptom onset (8, 20).Patients with PHO undergoing either surgical intervention or chemotherapy alone generally exhibit a survival duration of three months and a poor prognosis (10, 13, 18, 19, 22, 24).In comparison to PHO patients who did not receive combination therapy, PHO patients treated with combination therapeutics included targeted therapy, exhibited survival beyond six months, suggesting that combination therapeutics is essential for enhancing prognosis (17, 19, 28, 29). In osteosarcoma, KRAS mutations are key drivers of invasion, angiogenesis, metastasis and cell proliferation, hence affecting the disease’s aggressiveness. TKIs such as apatinib, lenvatinib and sorafenib, which target VEGFRs and RET, are viable therapeutic alternatives for osteosarcoma and may affect patient prognosis (36). The integrated results of the literature review demonstrated a survival advantage in three PHO patients treated with combination therapy (incorporating targeted therapy), who all survived beyond eight months. Compared with PHO patients not receiving combination therapy, these findings highlight that combination therapies are essential for improving prognosis (28, 29).

## 5 Conclusion

The median age of PHO is 61 years, and radiological identification of this entity relies on cystic-solid tumors, occasionally accompanied by calcifications. PHO seems to be more common in men. The pooled analysis of included studies indicates that TP53 gene mutations and aberrant SATB2 protein expression hold potential as molecular indicators for this illness. Despite the high malignancy and generally grim prognosis that PHO carries, the adoption of multifaceted treatment approaches including surgery, chemotherapy, and targeted therapy has demonstrated the potential to significantly improve survival rates.

## Data Availability

The datasets presented in this study can be found in online repositories. The names of the repository/repositories and accession number(s) can be found in the article/**Supplementary Material**.
